# Brain region specific mitophagy capacity could contribute to selective neuronal vulnerability in Parkinson's disease

**DOI:** 10.1186/1477-5956-9-59

**Published:** 2011-09-23

**Authors:** Madeleine Diedrich, Tohru Kitada, Grit Nebrich, Andrea Koppelstaetter, Jie Shen, Claus Zabel, Joachim Klose, Lei Mao

**Affiliations:** 1 Institute for Medical Genetics and Human Genetics, Charité Universitätsmedizin Berlin, D-13353 Berlin, Germany; 2 Center for Neurologic Diseases, Brigham and Women's Hospital, Program in Neuroscience, Harvard Medical School, Boston, MA 02115, USA

**Keywords:** Parkinson's disease, mitophagy, proteomics, PINK1, 2DE

## Abstract

Parkinson's disease (PD) is histologically well defined by its characteristic degeneration of dopaminergic neurons in the *substantia nigra pars compacta*. Remarkably, divergent PD-related mutations can generate comparable brain region specific pathologies. This indicates that some intrinsic region-specificity respecting differential neuron vulnerability exists, which codetermines the disease progression. To gain insight into the pathomechanism of PD, we investigated protein expression and protein oxidation patterns of three different brain regions in a PD mouse model, the PINK1 knockout mice (PINK1-KO), in comparison to wild type control mice. The dysfunction of PINK1 presumably affects mitochondrial turnover by disturbing mitochondrial autophagic pathways. The three brain regions investigated are the midbrain, which is the location of *substantia nigra*; striatum, the major efferent region of *substantia nigra*; and cerebral cortex, which is more distal to PD pathology. In all three regions, mitochondrial proteins responsible for energy metabolism and membrane potential were significantly altered in the PINK1-KO mice, but with very different region specific accents in terms of up/down-regulations. This suggests that disturbed mitophagy presumably induced by PINK1 knockout has heterogeneous impacts on different brain regions. Specifically, the midbrain tissue seems to be most severely hit by defective mitochondrial turnover, whereas cortex and striatum could compensate for mitophagy nonfunction by feedback stimulation of other catabolic programs. In addition, cerebral cortex tissues showed the mildest level of protein oxidation in both PINK1-KO and wild type mice, indicating either a better oxidative protection or less reactive oxygen species (ROS) pressure in this brain region. Ultra-structural histological examination in normal mouse brain revealed higher incidences of mitophagy vacuoles in cerebral cortex than in striatum and substantia nigra. Taken together, the delicate balance between oxidative protection and mitophagy capacity in different brain regions could contribute to brain region-specific pathological patterns in PD.

## 1 Introduction

Parkinson's disease (PD) is one of the most common neurodegenerative disorders in the elderly [1]. Approximately 4% of the population beyond age 65 years is affected by PD. An important clinical symptom of PD is impaired motor function manifested by resting tremor, rigidity, bradykinesia and postural instability [2]. One hallmark of PD is the selective degeneration of dopaminergic neurons in the *substantia nigra pars compacta*, one of the most important dopaminergic brain regions. Other brain regions, for instance cerebral cortex, are only affected in much advanced disease stages [3].

Although over 95% of the PD cases lack clear familial background [4], several recessive genetic mutations showing Mendelian inheritance pattern can lead to familial PD cases that demonstrate significant clinical overlaps with sporadic PD [5,6]. Such genetic hotspots include PARK2 (Parkin), PINK1, DJ-1, UCHL1, LRRK2 (leucine-rich repeat kinase 2) and PARK1 (alpha-synuclein) [4]. Most remarkably, all these very distinct mutations can generate rather indistinguishable PD pathology respecting brain region specific neuron loss. Moreover, several oxidative stress inducers such as MPTP (1-Methyl-4-phenyl-1, 2, 3, 6-tetrahydropyridin), 6-hydroxydopamine (6-OHDA) or paraquat, can faithfully reproduce comparable neuronal death pattern similar to that observed in PD [7-9]. This indicates that some intrinsic brain structural dynamics set the tune in PD progression.

Different hypotheses exist regarding this selective cell loss in PD, which refer to region specific neurotransmitter synthesis, neuromelanin formation or the divergent presence of neurotoxic substances [10,11]. Recently, it has become clear that mitochondrial dysfunction and oxidative damage represent a convergence point for PD and other neurodegenerative conditions [12]. In this respect, mutations in two familiar PD related genes, PARK2 (encoding Parkin) and PINK1 are both involved in the mitochondrial quality control machinery termed mitochondrial autophagy (also called mitophagy) [4,13]. However, this makes even more peculiar why such ubiquitously present mutations can lead to PD-specific selective neuron loss.

Based on the rationale that brain region specificity could be intensively monitored via differential protein expression profiles, we conducted an in-depth proteomic study in a PD mouse model, the PINK1 knockout mice (PINK1-KO) [4,14,15]. Our aim was to clarify the mechanism underlying the PD brain region specificity. PINK1, or PTEN-induced kinase 1, encodes a mitochondrial located kinase, the mutation of which shows high penetrance to early-onset PD [16-18]. Recently, its mode-of-action was elucidated in its involvement of mitophagy [19,20]. In our previous communication, we showed that the loss-of-function mutation of PINK1 leads to brain mitochondrial dysfunction and heightened susceptibility of neurons to oxidative stress [21]. Here, we analyzed the PINK1-KO mouse model by a two-dimensional difference gel electrophoresis (2D-DIGE) based approach to determine protein expression alterations in three distinct brain regions: the midbrain, which is the location of *substantia nigra*, the striatum, which is the major efferent region of *substantia nigra*, and the cerebral cortex, which is more distal, and thus less relevant to PD pathology. In addition, we used an immunostaining methodology to investigate the protein oxidation profiles in these different brain regions. Our results suggest that possibly, the differential mitophagy and oxidative protection capacity in different brain regions could contribute to region-specific neuronal death patterns in PD pathology.

## 2 Materials and methods

### 2.1 Animals and tissue samples

Animal experiments were carried out in accordance with the USA community guidelines and were approved by Harvard University Animal Care Committees. All efforts were made to minimize animal suffering and to reduce the number of animals used. Four-months-old PINK1-KO mice of 129Sv background (n = 6) and their wild type littermates (n = 6) were used for proteomic analyse (2D-DIGE and Oxyblot). Three additional wild type mice were used for electron microscopic investigations. For the generation of PINK-KO mice, PINK-KO embryonic stem cells were injected into blastocytes of C57BL/6 mice. Chimeric mice obtained were intercrossed with (129/Sv × C57BL/6)F1 hybrid mice to acquire heterozygous mutant mice, which were then intercrossed to obtain homozygous mutant mice of 129Sv background. These obtained PINK1^-/- ^mice (termed PINK-KO in the manuscript) were kept in the 129/Sv background. The ventral midbrain and the dorsal striatum tissues were dissected out surgically. After removing brain stem and cerebellum of the brain, we used the remaining portion (forebrain) as cortex samples.

### *2*.2 Sample preparation for two-dimensional electrophoresis

Total protein extracts of mouse striatum, midbrain and cortex tissues were prepared for each sample separately as described previously [22]. Briefly, about 25 mg of the tissue was ground in an Eppendorf tube submersed in liquid nitrogen, adding 1.8 parts (v/w) of 50 mM Tris buffer (pH 7.5) containing 50 mM KCl, 20% (v/v) glycerol, 4% (w/v) 3-[(3-cholamidopropyl) dimethyl-ammonio]-1-propane sulfonate (CHAPS), a phosphatase inhibitor mixture (PhosStop, Roche, 04906845001) and a protease inhibitor cocktail (Complete™, applied according to manufacturers instruction, Roche, 11697498001). Finally, glass beads (1.5-1.7 mm diameter, Wolf Glaskugeln GmbH) equivalent to 0.034 times of sample weight were added to the mixture and samples were sonicated six times (20 seconds each, 1 minute interval under stirring) in an ice-water bath (0°C). The resulting tissue homogenate was stirred for 30 minutes at 4°C after adding 0.025 parts (v/v) Benzonase (Novagen, 70746). Subsequently, 6.5 mol/L urea and 2 mol/L thiourea were added to the sample. Protein concentration was determined using the Roti-Nanoquant Kit (Roth, K880.1). Samples were stored at -80°C until subsequent analysis.

### 2.3 Two dimensional difference gel electrophoresis (2D-DIGE) and protein expression pattern evaluation

80 μg protein of experimental (wild type control or PINK1-KO) and the pooled reference samples were labeled with fluorescence dye Cy5 or Cy3, respectively, according to the manufacturer instructions (CyDye DIGE Flours minimal dyes, GE Healthcare, 25-8008-61 and 25-8008-62). As an internal standard for the DIGE quantification system, the pooled reference sample consists of 6.67 μg of protein extract from each of six control and six PINK1-KO mouse midbrain samples [23]. Proteins were separated by 2D-DIGE as described previously [22,24]. In short, capillary tube gels (40 cm in length, 1.5 mm diameter) for isoelectric focussing were prepared with a special mixture of carrier ampholytes covering a pH range of 3.5 to 9.5. The SDS-PAGE gel format was 40 cm × 30 cm. The gels were scanned using a Typhoon 9400 laser scanner (GE Healthcare). Image analysis was performed with the Delta2D software (version 3.4 and 4.0, DECODON). 16 bit grey scale images were imported into the program. Delta2D contains a special feature to handle DIGE experiments which considers the reference data for protein concentration normalization and quantification. A fusion image over the whole experiment was generated and employed for spot detection. After manual spot editing to eliminate inaccurate spots, the spot pattern was transferred to all gel images within the project. The signal intensities of each spot were computed as a weighted sum of all pixel intensities ("volume" of protein spot). The total pixel amount of all detected spots on the parental gels was taken as 100%. This allows the software to deduce the relative spot intensity of each individual spot as percentage. In turn, this relative spot intensity data, which are a quasi-linear correlates to protein concentration, were used to access the protein expression alterations in PINK1-KO mouse. Here, over ninety-five percent of the protein spots on the 2D gels that did not vary in their spot intensity served as reference for normalization. Normalized values after local background subtraction were subsequently exported from Delta2D in spreadsheet format. Unpaired Student's T-Tests were used to determine significant protein spot differences between the PD mouse model and controls (p < 0.05). False discovery rate (FDR) of less than 5% was controlled using the Delta2D software in-build permutation based statistics. Only expression changes over 20% were considered for further analysis.

### 2.4 Protein identification by mass spectrometry

Database-assisted protein identification using mass spectrometry observed the up-to-date guidelines [25]. 500 μg protein extract from each brain region was used as described previously [26]. Briefly, proteins were separated by two-dimensional protein electrophoresis and visualized using a MS-compatible silver staining protocol [27]. Protein spots-of-interest were excised from the 2DE gel and in-gel trypsin digestion was performed. Tryptic fragments were analyzed either by a Reflex IV MALDI-TOF mass spectrometer (Bruker Daltonics) or LCQ Deca XP nanoHPLC/ESI ion trap mass spectrometer (Thermo Fisher Scientific). Mass spectra were analyzed using in-house licensed Mascot software (version 2.1, Matrix Science) by automatic searches in NCBI non-redundant databases restricted to taxonomy *Mus musculus*. Search parameters allowed for one miscleavage and oxidation of methionine and propionamidation of cysteine. Criteria for positive identification of proteins with mass spectrometry were set according to the probability based Mowse score algorithm (p ≤ 0.05) [28]. For protein identification using ESI-MS/MS, a minimum of two peptides was set as an additional criterion of successful protein identification.

### 2.5 Protein functional categorization

In order to investigate the functional impact of protein expression alterations induced by PINK1 knockout, variant proteins detected in this study were subjected to functional characterization with the help of public databases. This includes Mouse Genome Informatics (MGI) and Webgestalt http://bioinfo.vanderbilt.edu/webgestalt/, which conduct gene set enrichment analyses by cross-referencing downstream public databases including Gene Ontology (GO, http://www.geneontology.org/) and Kyoto Encyclopedia of Genes and Genomes (KEGG, http://www.genome.jp/kegg/). Hereby, the entire mouse (Mus musculus) proteins were used as a reference set. To ensure the statistical significance of the enrichment analysis, the hypergeometric test was used with the statistical threshold p < 0.0001, while the Bonferroni-Holm method served as the multiple test adjustment control. The adjusted p-values were reported in the result section, as these are more stringent compared to the raw p-values. A minimum of three genes was set as an additional cut-off for the enrichment analyses.

### 2.6 Immunostaining for oxidized proteins (Oxyblot)

Oxidized proteins were detected by specific antibody against protein carbonyl moieties on protein side chains after Western blotting. For this purpose, precisely 100 μg protein extract each were used from pooled controls (n = 6) and pooled transgenic samples (n = 6) of midbrain, striatum or cortex. Experiments were repeated three times with identical treatment. This constitutes a basis for the subsequent quantification procedure of the oxidized proteins.

Samples were first separated by 2DE with the gel format 16 cm (isoelectric focusing) × 12 cm (SDS-PAGE) [22,29]. Subsequently, the proteins were transferred to a PVDF-membrane (Millipore, IPVH00010) using the Trans-Blot^® ^SD semi-dry electrophoretic transfer cell (100 mA, 150 min, Bio-Rad). The membranes were blocked overnight at 4°C with 5% skim dry milk solution in TBST buffer (containing 0.1% Tween 20) under mild agitation. The Oxyblot immunostaining was performed according to the manufacture's instruction (OxyBlot™ Protein Oxidation Detection Kit, Millipore, S7150). In an incubation step, carbonyl groups on the protein side chains were subjected to a condensation reaction with 2, 4-dinitrophenylhydrazine to yield 2, 4-dinitrophenylhydrazone (DNP-hydrazone). Subsequently, oxidized proteins were immunodetected using an antibody specific to the DNP-hydrazone moieties of the proteins. For chemical luminescence imaging, appropriate horseradish peroxidase-conjugated secondary antibody was applied (Amersham™, anti-rabbit, NA934V and ECL™kit, GE Healthcare, RPN2106). Exactly 15 seconds of exposure time was applied for each Oxyblot development. Based on the identical expression setting and the same amount of proteins applied on each Oxyblot experiment, the fluorescent signal intensity was used to compare the amount of oxidized protein level among different samples. The extent of overall protein oxidation of each sample was quantified using ImageJ http://rsbweb.nih.gov/ij. For the statistical data evaluation, we first compared the total protein oxidation extend of PINK1-KO to wild type controls in each of the three brain regions (striatum, midbrain and cortex) using Student's T-test. Given the lack of significant difference between PINK1-KO and control, we combined values of these two groups (PINK1-KO and control) of each mouse brain region, and investigated protein oxidation level in regard to different brain regions. For this purpose, ANOVA was first performed to detect among-group difference (p < 0.05), whereas unpaired Student's T-test was used subsequently to assess between-group difference (p < 0.05).

### 2.7 Electron microscopy of mouse brain regions

For the electron microscopy of different brain regions, cortex, striatum and substantia nigra (SN) tissue samples of about one cubic mm of size were obtained from normal control mice (n = 3). Notice that we used *substantia nigra *instead of the whole midbrain tissue, as this is the most vulnerable region in PD pathology. Samples were immediately submerged in 2.5% glutaraldehyde solution in PBS for 16 hours. Electron microscopic analysis was carried out on Epon embedded ultrathin tissue slides as previously described [30,31]. The presences of total of mitochondria, defective mitochondria (showing swelling and disintegration of cistenea), mitochondria under mitophagic process (mitophagic vacuoles), as well as mitochondria under fission and/or fusion process were quantified according to 30 random micrographs for each tissue sample (magnification 3597 to 10, 000). Quantitative data of total mitochondria, defective mitochondria were assessed regarding to their numbers per cubic micrometer [(μm^2)^-1^] on original tissue samples. Unpaired Student's T-test was used to assess the statistical significance of the value in different brain regions.

## 3 Results

### 3.1 Number and identity of protein alterations in PINK1-KO mice are brain region specific

**Figure 1:**
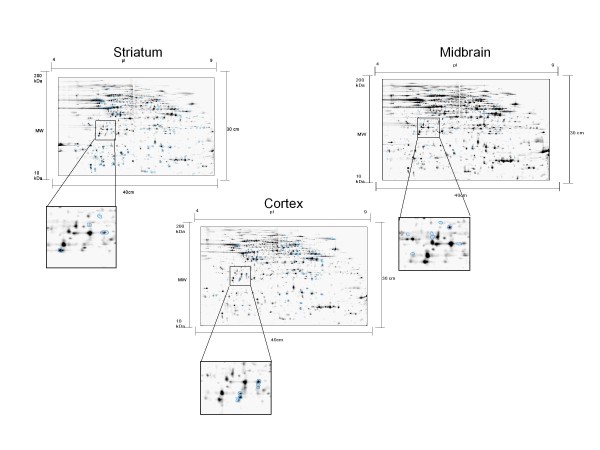
**Representative two-dimensional protein expression patterns of three different brain regions (striatum, midbrain and cortex) showing significantly altered protein spots (blue circled, unpaired student T-test, n = 6, p < 0.05. Over 20% of expression alteration)**. Over 5000 protein spots could be detected on each protein pattern. The gel insets demonstrate that there was little overlap of altered protein isoforms among the three brain regions (gel images from PINK1-KO mice).

Protein expression alterations induced by PINK1 knockout were accessed by the quantitative comparison of the 2DE patterns of PINK1-KO mice (n = 6) to normal control mice (n = 6) in three brain regions. In general, over 5000 protein spots could be detected on each of the 2DE protein patterns. However, protein expression alterations of each brain region in the PD mouse model were quite unique respecting their isoelectric point and molecular weight distribution (Figure 1). Among the three brain regions investigated, the striatum tissue showed the highest overall number of protein expression alterations (Table 1). Compared to wild type mice, 95 protein isoforms were up-regulated and 78 were down-regulated in striatum of PINK1-KO mice. The total number of protein alterations in striatum was over two-fold higher than in the cortex and three-fold higher in regard to midbrain. One hundred protein spots among these variant proteins (58%) could be successfully identified with mass spectrometry. Unexpectedly, these protein spots were assigned to merely 20 protein encoding genes. This suggests that PINK1-KO induced protein alteration in striatum represents predominantly at the post translational modification level. In midbrain tissue, which is the location of *substantia nigra*, we detected a total of 62 protein spots that were differentially expressed in PINK1-KO mice. The majority of them (51) were down-regulated, whereas 11 protein isoforms were up-regulated. Out of these 62 protein spots, 57 (92%) could be identified, which yield 30 distinct protein encoding genes. In cortex tissue samples of the PINK1-KO mice, 75 protein spots were found to be up-regulated while seven proteins were down-regulated. Eighty-five percent (70/82) of these altered proteins could be identified and were assigned to 28 non-redundant gene symbols.

**Table 1:** Number of differentially expressed proteins and protein isoforms in striatum, midbrain and cortex tissues of PINK1-KO mice compared to wild-type mice

Brain region	Differentially expressed protein isoforms*	Identified protein isoforms	Number of corresponding genes
**striatum**	173 (95↑, 78↓)	100	20

**midbrain**	62 (11↑, 51↓)	57	30

**cortex**	82 (75↑, 7↓)	70	28

Over 5000 protein spots were investigated (Student's t-test, p < 0.05, changes over 20% vs. wildtype control).

*: ↑, up-regulated; ↓, down-regulated.

**Figure 2:**
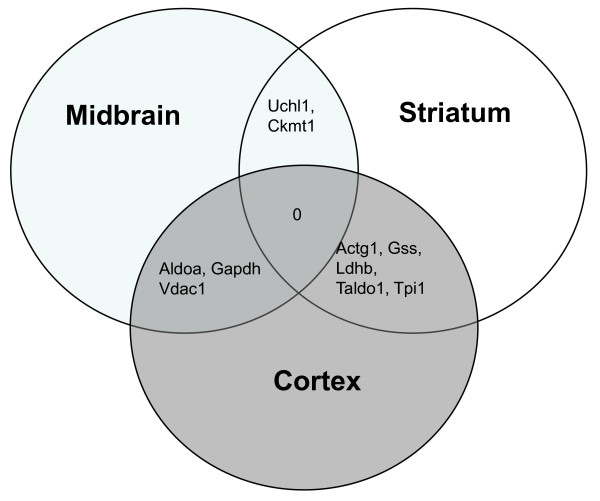
**Expression alteration overlaps among three brain regions in PINK1-KO mice were studied at the level of gene names**. Concomitant protein alterations were observed only between two brain regions at a time. The regulatory pattern of expression (up- or down-regulation) was not taken into account.

In a careful comparison of all three brain regions respecting their protein expression alterations in PINK1-KO mice, no single protein was observed to be altered in all three brain regions. However, two proteins (Uchl1 and Ckmt1) were co-changed in striatum and midbrain tissues of PINK1-KO mice (although of bifurcated direction), whereas five proteins were co-altered in striatum and cortex tissues (Actg1, Gss, Ldhb, Taldo1 and Tpi1). When comparing midbrain vs. cortex, three proteins showed concomitant regulations. They are Aldoa, Gapdh and Vdac1 (Figure 2). An overview of the number of identified protein expression alterations in all three brain regions is given in table 1.

### 3.2 PINK1 knockout exerted brain region specific impact on mitochondrial proteins

Altered proteins in PINK1-KO mice were clustered according to their functional involvement and cellular localization. Respecting their cellular component identity, the up-regulated proteins in PINK1-KO mouse brain tissues were enriched in the GO-terms "mitochondrial membrane" (p = 5.48e-05; Atp5c1, Ckmt1, Mdh2, Vdac1, Idh2, Dhrs1, Suclg1) and "actin filament" subunits (p = 1.23e-3; Actb, Actg1). The protein subsets of down-regulated proteins in PINK1-KO showed enrichments in the GO-terms "mitochondria" (p = 3.78e-08; Ldha, Cs, Got2, Ak3, Vdac3, Pdhb, Mecr, Ckmt1, Gapdh, Etfa, Slc25a12, Vdac1, Glod4, Mdh1).

Cellular functional involvement analysis on altered proteins in PINK1-KO mice showed significant overrepresentation of various energy metabolism pathways, with partially divergent regulation directions in different brain regions. KEGG pathways that bear significant overrepresentation in altered proteins in PINK1-KO mice are summarized in table 2. A common perturbed pathway in all three brain regions is glycolysis and gluconeogenesis. In cortex tissue of the PINK1-KO mice, six proteins (Aldoa, Eno1, Gapdh, Ldhb, Pgk1 and Tpi1) belonging to this pathway were up-regulated. In contrast, five proteins participating in glycolysis and gluconeogenesis pathway were down-regulated in the midbrain tissue of our PD mouse model (Aldoa, Eno2, Gapdh, Ldha and Pdhb). In addition, proteins of the Krebs cycle, which is downstream to glycolysis, showed increased expression in striatum region of the PINK1-KO mice. Similarly, there were indications that mitochondrial respiration chain proteins were significantly down-regulated in midbrain, but up-regulated in striatum and cortex. For example, the electron transfer flavoprotein subunit alpha (Etfa) was down-regulated in midbrain tissue of PINK1-KO mice compared to that of wild type mice. In contrast, one subunit of ATP synthase (Atp5c1) was drastically (2.2-fold) up-regulated in cortex tissues of the PINK1-KO mice. Apart from energy metabolism, we observed a consistent down regulation of three protein isoforms of mitochondrial channel protein Vdac1 and one isoform of Vdac3 in the midbrain tissue of PINK1-KO mice. In contrast, Vdac1, the essential membrane potential keeper of mitochondria, was 47% up-regulated in cortex tissue.

**Table 2:** Functional pathways (KEGG) that were overrepresented in PINK1-KO induced protein expression alterations of different brain regions

*Up-regulated proteins in striatum:*		
**KEGG pathway**	**Number of proteins**	**Multiple test adjusted p-value**

Metabolic pathways	Sucla2, Mdh2, Ckb, Gss, Idh2, Echs1, Ckmt1, Tpi1, Ldhb, Aldoc, Taldo1	2.66 e-13

Citrate cycle (TCA cycle)	Sucla2, Mdh2, Idh2	4.02E-07

Propanoate metabolism	Sucla2, Ldhb, Echs1	4.02E-07

Glycolysis/Gluconeogenesis	Tpi1, Ldhb, Aldoc	1.42E-05

***Down-regulated proteins in midbrain:***		

**KEGG pathway**	**Number of proteins**	**Multiple test adjusted p-value**

Metabolic pathways	Ldha, Aldoa, Eno2, Pdhb, Mecr, Ckmt1, Suclg1, Gapdh, Pafah1b2, Mdh1	3.30 e-10

Glycolysis/Gluconeogenesis	Gapdh, Ldha, Aldoa, Eno2, Pdhb	4.40E-09

Citrate cycle (TCA cycle)	Suclg1, Pdhb, Mdh1	8.11E-07

Pyruvate metabolism	Ldha, Pdhb, Mdh1	1.44E-06

***Up-regulated proteins in cortex:***		

**KEGG pathway**	**Number of proteins**	**Multiple test adjusted p-value**

Metabolic pathways	Cs, Auh, Aldoa, Gss, Abat, Atp5c1, Pgk1, Eno1, Tkt, Gapdh, Tpi1, Ldhb, Nme1, Got1, Taldo1	4.28E-18

Glycolysis/Gluconeogenesis	Pgk1, Eno1, Gapdh, Tpi1, Ldhb, Aldoa	6.93E-11

Pentose phosphate pathway	Tkt, Aldoa, Taldo1	1.55E-06

Regulation of actin cytoskeleton	Actg1, Actb, Pfn1	3.00E-04

### 3.3 Cerebral cortex tissue showed higher basal and dynamic levels of oxidative protection

Indications of selective oxidative protection were observed among different brain regions in our mouse bearing PINK1 loss-of-function. This was reflected predominantly by the behavior of the cellular antioxidant synthesizer glutathione synthetase (Gss). This enzyme was 42% up-regulated in the striatum, and 24% up-regulated in the cortex tissue of PINK1-KO mice. Consistent with this finding, peroxiredoxin 1 (Prdx1) protein was slightly up-regulated in cortex tissue of the transgenic mice. In contrast, no change of these antioxidant proteins was observed in midbrain tissues.

**Figure 3:**
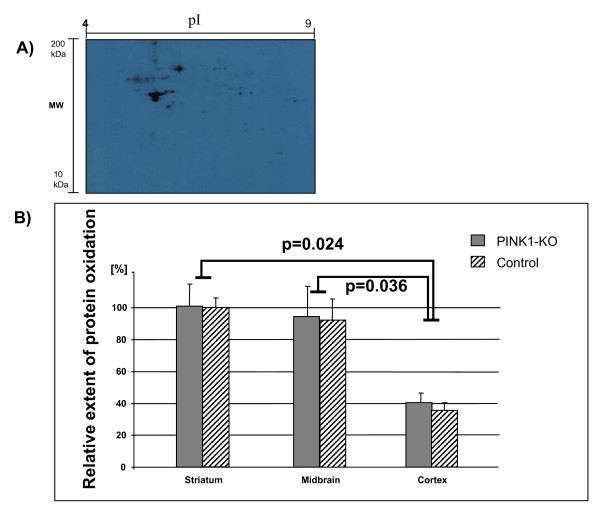
**Immunostaining of protein carbonyl moieties revealed significantly lower amount of oxidized proteins in cortex tissue than in striatum and midbrain in both PINK1-KO and wild type mice (ANOVA analysis followed by unpaired Student T-test, p < 0.05)**. A) A representative Oxyblot image of a 2DE gel immunostained against protein carbonyls (image from PINK1-KO, midbrain). B) Bar chart showing differential protein oxidation extents in striatum, midbrain and cortex tissues of PINK1-KO and control mice (mean ± SEM, n = 3. see Materials and Methods for detail). Hereby, the average signal intensity of protein oxidation in striatum of control mice was set as 100%. Experiments were repeated three times on exactly 100 μg of pooled protein extract of each brain region.

In order to detect the extent of oxidative damage in mouse proteome, we employed a well established methodology of redox proteomics on the 2DE gels (Figure 3A) [32]. Contrary to our expectation, comparison of PINK1-KO mouse to control samples respecting total amount of oxidized proteins (protein carbonyls) revealed no significant differences. However, when comparing the three brain regions to each other, cerebral cortex tissue showed a significantly lower degree (ca. 60%) of oxidized proteins than midbrain or striatum. When using wild type mouse striatum tissue as an internal reference (the mean value of which was set as 100%), the amount of oxidized proteins in midbrain accounts 94.5% ± 19%. However, a significant fewer amounts (40.4% ± 6%) of the proteins were oxidized in cortex tissues compared to either midbrain (p = 0.036) or striatum tissues (p = 0.024). As shown in Figure 3B, this finding was obviously independent of the PINK1 knockout.

### 3.4 Lack of feedback stimulation of alternative catabolic programs in midbrain

Since the direct mode-of-action of PINK1 dysfunction is the disturbance of cellular mitophagy, we set out to investigate mitophagy-related protein expression alteration as well as other catabolic processes in different brain regions of PINK1-KO mice. In this respect, we observed that the heat shock protein 70 (Hspa8), an important player of chaperone-mediated autophagy, was significantly up-regulated in cortex tissue, but not in striatum or midbrain. Regarding cellular catabolic programs other than autophagy, ubiquitin carboxy-terminal hydrolase L1 (Uchl1) was significantly down-regulated in midbrain, but up-regulated in striatum tissue of the PINK1-KO mice. Two other proteins that are involved in the ubiquitination pathway were also significantly down-regulated in midbrain (Uba1, Ulip3). Contrary to this, two isoforms of the Ulip3 protein were observed to be up-regulated in the cerebral cortex tissue of PINK1-KO mice. Notably, no up-regulation of such catabolic process related proteins was observed in midbrain tissues of the PD mouse model.

Detailed information regarding protein identification and expression pattern is provided in Table 3.

**Table 3:** Detailed information regarding protein identification and expression pattern in striatum, midbrain and cortex tissues of the PINK1-KO (KO) mice in respect to wildtype control mice (WT):

Striatum:											
**NCBI access number**	**Gene symbol**	**Number of isoforms**	**Protein name**	**Expression ratio ** **(KO vs. wt)**	**T test (p-value)**	**Mowse score**	**Sequence coverage (%)**	**Number of matched peptides**	**Molecular weight (Da)**	**Theoretical pI**	**Functional annotation (KEGG pathway)**

gi|6753428	Ckmt1	1	creatine kinase, mitochondrial 1, ubiquitous	1.37	0.0002	235	12	5	46974	8.39	Metabolic pathways; Arginine and proline metabolism

gi|6755929	Uchl1	7	ubiquitin carboxy-terminal hydrolase L1	1.45	0.0008	67	47	9	24822	5.33	Parkinson's disease

gi|3766201	Sucla2	4	ATP-specific succinyl-CoA synthetase beta subunit	1.42	0.001	299	21	6	46215	5.65	Metabolic pathways; Citrate cycle (TCA cycle); Propanoate metabolism;

gi|6678674	Ldhb	5	lactate dehydrogenase 2, B chain	1.37	0.0012	136	51	2	36549	5.7	Metabolic pathways; Propanoate metabolism; Glycolysis/Gluconeogenesis; Pyruvate metabolism;

gi|31980844	Dhrs1	2	dehydrogenase/reductase (SDR family) member 1	1.54	0.0016	395	21	7	33983	8.66	

gi|10946574	Ckb	5	creatine kinase, brain	1.42	0.0018	465	25	8	42686	5.4	Metabolic pathways; Arginine and proline metabolism

gi|112363107	Nefm	7	Neurofilament triplet M protein	0.43	0.0025	56	12	2	95984	4.76	Amyotrophic lateral sclerosis (ALS)

gi|6680117	Gss	3	glutathione synthetase	1.42	0.0035	436	16	8	52214	5.56	Metabolic pathways; Glutathione metabolism;

gi|15277976	Ndrg2	3	N-myc downstream regulated gene 2	1.4	0.0046	93	6	2	40763	5.23	

gi|809561	Actg1	4	gamma-actin	1.32	0.0048	84	5	2	40992	5.56	Regulation of actin cytoskeleton

gi|387422	Mdh2	8	malate dehydrogenase	1.37	0.0079	222	12	4	35588	8.93	Metabolic pathways; Citrate cycle (TCA cycle); Pyruvate metabolism;

gi|14198249	Aldoc	4	Fructose-bisphosphate aldolase C	1.34	0.0097	144	9	3	39307	6.47	Metabolic pathways; Glycolysis/Gluconeogenesis; Pentose phosphate pathway; Fructose and mannose metabolism

gi|13097102	Ddah2	2	dimethylarginine dimethylaminohydrolase 2	1.32	0.0098	183	18	4	29627	5.66	

gi|33859640	Taldo1	3	transaldolase 1	1.34	0.0109	163	12	4	37363	6.57	Metabolic pathways; Pentose phosphate pathway;

gi|6681195	Dlg4	4	postsynaptic density protein 95	0.64	0.0133	38	4	2	80423	5.56	Huntington's disease

gi|6753476	Cnp	1	cyclic nucleotide phosphodiesterase 1	1.42	0.0142	371	21	8	47094	9.08	

gi|27370516	Idh2	6	isocitrate dehydrogenase 2 (NADP+), mitochondrial	1.37	0.0151	221	10	4	50902	8.88	Metabolic pathways; Citrate cycle (TCA cycle); Glutathione metabolism;

gi|54855	Tpi1	25	triosephosphate isomerase	1.36	0.0214	770	42	12	26679	6.9	Metabolic pathways; Glycolysis/Gluconeogenesis; Fructose and mannose metabolism;

gi|12805413	Echs1	2	Echs1 protein	1.31	0.0245	428	26	8	31237	8.76	Metabolic pathways; Propanoate metabolism;

gi|7305485	Sh3gl1	4	SH3-domain GRB2-like 1	1.28	0.0307	136	7	3	41492	5.53	Endocytosis

**Midbrain:**											

**NCBI access number**	**Gene symbol**	**Number of isoforms**	**Protein name**	**Expression ratio (KO vs. wt)**	**T test (p-value)**	**Mowse score**	**Sequence coverage (%)**	**Number of matched peptides**	**Molecular weight (Da)**	**Theoretical pI**	**Functional annotation (KEGG pathway)**

gi|13384652	Mecr	1	trans-2-enoyl-CoA reductase, mitochondrial precursor	0.53	0.001	88	4	2	40316	9.34	Metabolic pathways;

gi|6679937	Gapdh	3	similar to glyceraldehyde-3-phosphate dehydrogenase	0.55	0.002	340	19	5	35787	8.44	Metabolic pathways; Glycolysis/Gluconeogenesis;

gi|12849397	Glod4	1	Glyoxalase domain-containing protein 4	0.69	0.003	545	46	11	33296	5.28	

gi|6753428	Ckmt1	2	creatine kinase, mitochondrial 1, ubiquitous	0.77	0.003	123	14	2	46974	8.39	Metabolic pathways;

gi|18017596	Snx4	1	sorting nexin 4	0.39	0.007	140	6	2	51745	5.58	

gi|27369581	Slc25a12	1	solute carrier family 25 (mitochondrial carrier, Aralar), member 12	0.46	0.007	318	9	5	74523	8.43	

gi|21410877	Rap1gap	1	Rap1gap protein	0.72	0.007	62	9	2	45618	5.47	

gi|387129	Mdh1	3	cytosolic malate dehydrogenase	0.74	0.01	145	11	3	36454	6.16	Metabolic pathways; Citrate cycle (TCA cycle); Pyruvate metabolism;

gi|33440467	Pafah1b2	2	Platelet-activating factor acetylhydrolase, isoform 1b, alpha2 subunit	0.72	0.015	91	8	2	25476	5.57	Metabolic pathways;

gi|21759113	Etfa	2	Electron transfer flavoprotein subunit alpha, mitochondrial precursor (Alpha-ETF)	0.61	0.016	455	29	6	35018	8.42	

gi|1915915	Ulip3	1	Ulip3 protein	0.73	0.016	177	12	3	62142	6.39	

gi|31981086	Efhd2	3	EF hand domain containing 2	0.66	0.018	602	45	9	25084	5.07	

gi|18606238	Gsn	1	gelsolin	0.7	0.018	328	9	5	80712	5.83	Regulation of actin cytoskeleton

gi|6671539	Aldoa	4	aldolase 1, A isoform	0.79	0.019	1158	59	17	39331	8.31	Metabolic pathways; Glycolysis/Gluconeogenesis;

gi|6678483	Uba1	1	ubiquitin-activating enzyme E1, Chr X	0.76	0.021	669	13	10	117734	5.43	Parkinson's disease

gi|18152793	Pdhb	1	pyruvate dehydrogenase (lipoamide) beta	0.78	0.021	497	32	11	38912	6.41	Metabolic pathways; Glycolysis/Gluconeogenesis; Citrate cycle (TCA cycle); Pyruvate metabolism;

gi|6755963	Vdac1	3	voltage-dependent anion channel 1	0.66	0.0217	855	51	10	30737	8.62	Parkinson's disease; Huntington's disease; Calcium signaling pathway

gi|6755967	Vdac3	1	voltage-dependent anion channel 3	0.66	0.025	224	20	4	30733	8.96	Parkinson's disease; Huntington's disease; Calcium signaling pathway

gi|2690302	Got2	2	aspartate aminotransferase precursor	0.77	0.027	533	23	8	47382	9.05	Metabolic pathways; Alanine, aspartate and glutamate metabolism

gi|11141704	Sir2L2	1	sirtuin 2	0.48	0.029	51	4	2	43244	8.22	

gi|22902419	Gpd1l	2	glycerol-3-phosphate dehydrogenase 1-like	0.48	0.029	61	4	2	42517	8.22	Glycerophospholipid metabolism

gi|7106301	Mapre1	1	microtubule-associated protein, RP/EB family, member 1	0.56	0.031	193	18	3	29997	5.12	

gi|8567410	Syn2	1	synapsin II	0.61	0.034	70	3	2	52418	7.62	

gi|6671569	Arbp	2	acidic ribosomal phosphoprotein P0	1.41	0.037	196	19	4	34195	5.91	

gi|9845299	Suclg1	1	succinate-CoA ligase, GDP-forming, alpha subunit	1.21	0.039	88	9	2	34953	9.45	Metabolic pathways; Citrate cycle (TCA cycle); Propanoate metabolism

gi|61098212	Uchl1	1	ubiquitin carboxy-terminal hydrolase L1	0.65	0.04	157	16	3	24822	5.14	Parkinson's disease

gi|6754524	Ldha	6	lactate dehydrogenase 1, A chain	0.61	0.046	151	14	3	36475	7.62	Metabolic pathways; Glycolysis/Gluconeogenesis; Pyruvate metabolism; Propanoate metabolism

gi|29789104	Napb	1	N-ethylmaleimide sensitive fusion protein attachment protein beta	0.76	0.046	708	42	10	33536	6.41	

gi|7305027	Eno2	4	enolase 2, gamma neuronal	0.69	0.047	861	43	13	47267	4.99	Metabolic pathways; Glycolysis/Gluconeogenesis;

gi|55931021	Gdi2	3	Gdi2 protein	1.64	0.049	158	13	3	50506	5.93	

**Cortex:**											

**NCBI access number**	**Gene symbol**	**Number of Isoforms**	**Protein name**	**Expression ratio (KO vs. wt)**	**T test (p-value)**	**Mowse score**	**Sequence coverage (%)**	**Number of matched peptides**	**Molecular weight (Da)**	**Theoretical pI**	**Functional annotation (KEGG pathway)**

gi|4760600	Ak3	1	adenylate kinase isozyme 3	0.66	0.0004	124	47	11	24625	8.57	

gi|13385942	Cs	5	citrate synthase	0.74	0.0057	130	28	16	51703	8.72	Metabolic pathways;

gi|37700232	Nme1	4	nucleoside-diphosphate kinase 1	1.22	0.0006	91	48	9	17197	6.84	Metabolic pathways;

gi|19547889	Gss	3	glutathione synthetase	1.24	0.0386	107	26	12	51913	6.52	Metabolic pathways;

gi|42542422	Hspa8	5	Heat shock protein 8	1.24	0.0019	180	35	23	68074	5.32	

gi|123230136	Prdx1	1	peroxiredoxin 1	1.24	0.0228	64	23	5	18915	6.82	

gi|37202121	Abat	3	4-aminobutyrate aminotransferase	1.25	0.0005	227	37	24	56416	8.35	Metabolic pathways; Alanine, aspartate and glutamate metabolism; Propanoate metabolism; Valine, leucine and isoleucine degradation

gi|113680348	Fscn1	2	fascin homolog 1, actin bundling protein	1.26	0.0017	168	42	17	54474	6.44	

gi|34784434	Eno1	4	Eno1 protein	1.26	0.0062	139	43	16	39757	5.86	Metabolic pathways; Glycolysis/Gluconeogenesis;

gi|56789289	Ldhb	4	Ldhb protein	1.27	0.0011	86	42	7	11317	5.73	Metabolic pathways; Glycolysis/Gluconeogenesis; Propanoate metabolism; Cysteine and methionine metabolism

gi|6679439	Ppia	4	peptidylprolyl isomerase A	1.3	0.0006	99	53	12	17960	7.74	

gi|6755040	Pfn1	3	profilin 1	1.31	0.0022	176	73	14	14948	8.46	Regulation of actin cytoskeleton

gi|6678469	Tuba1c	2	tubulin, alpha 1C	1.32	0.0000088	98	35	13	49877	4.96	

gi|809561	3	1	gamma-actin	1.32	0.0028	230	56	24	41724	5.3	Regulation of actin cytoskeleton

gi|6679937	Gapdh	6	glyceraldehyde-3-phosphate dehydrogenase	1.33	0.0005	211	60	23	35787	8.44	Metabolic pathways; Glycolysis/Gluconeogenesis;

gi|49868	Actb	2	beta-actin (aa 27-375)	1.35	0.0019	237	54	27	39161	5.79	Regulation of actin cytoskeleton

gi|148686116	Taldo1	2	transaldolase 1, isoform CRA_e	1.39	0.0008	156	51	18	31514	7.66	Metabolic pathways; Pentose phosphate pathway

gi|6678413	Tpi1	1	triosephosphate isomerase 1	1.39	0.0006	147	42	9	26696	6.9	Metabolic pathways; Glycolysis/Gluconeogenesis; Fructose and mannose metabolism

gi|123210063	---	1	novel protein	1.44	0.0009	38	20	4	24445	8.76	

gi|6755963	Vdac1	1	voltage-dependent anion channel 1	1.47	0.0019	161	62	15	30737	8.62	

gi|6671539	Aldoa	4	aldolase 1, A isoform	1.52	0.005	179	43	17	39331	8.31	Metabolic pathways; Glycolysis/Gluconeogenesis; Pentose phosphate pathway; Fructose and mannose metabolism

gi|1915915	Dpysl1	2	Ulip3 protein	1.56	0.0058	225	40	24	62142	6.39	

gi|20072952	Auh	2	Auh protein	1.62	0.000068	114	32	13	32621	9.57	Metabolic pathways; Valine, leucine and isoleucine degradation

gi|11066098	Tkt	1	transketolase	1.89	0.0058	65	26	11	60545	6.54	Metabolic pathways; Pentose phosphate pathway;

gi|21311871	Nebl	1	nebulette	2.2	0.0007	74	40	13	31093	8.54	

gi|163838648	Atp5c1	1	ATP synthase, H+ transporting, mitochondrial F1 complex, gamma subunit isoform b	2.2	0.0007	126	42	15	30237	8.86	Metabolic pathways;

gi|202423	Pgk1	2 (with divergent regulation)	phosphoglycerate kinase	1.54; 0.77	5.00E-03; 1.22E-02	265	58	28	44522	8.02	Metabolic pathways; Glycolysis/Gluconeogenesis;

gi|160298209	Got1	2 (with divergent regulation)	glutamate oxaloacetate transaminase 1, soluble	1.62; 0.78	1.12E-02; 2.60E-03	130	30	12	46219	6.68	Metabolic pathways; Alanine, aspartate and glutamate metabolism

### 3.5 Brain region specific mitophagy capacity in wild type mice

**Figure 4:**
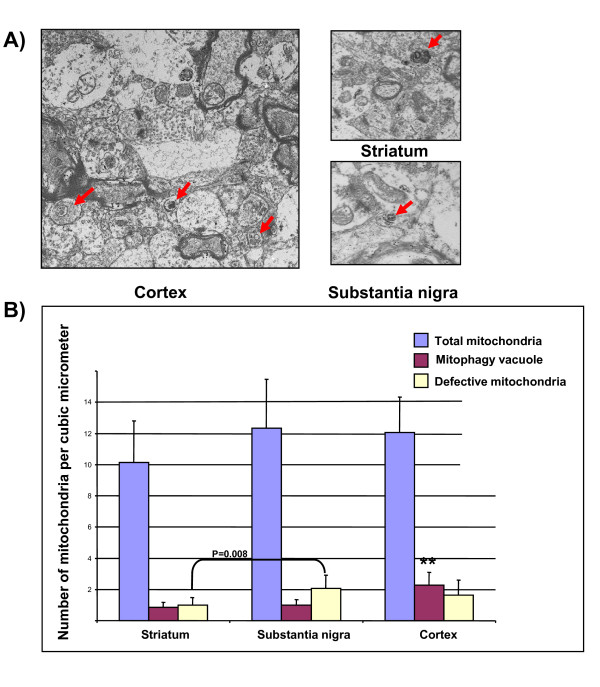
Electron microscopy revealed higher incidences of mitophagic vacuoles in cortex tissue of wild type mouse (designated as ** in figure. ANOVA test followed by unpaired student T-test, n = 30 for each brain region, p < 0.05). A) Insets of electron micrographs highlighting typical mitophagic vacuoles (arrow heads) that contain mitochondria and other cargos found in the cortex, striatum and *substantia nigra*. B) Bar chart showing the number of total mitochondria, mitophagic vacuoles and defective mitochondria in three mouse brain regions investigated (mean ± SEM). The cortex, striatum and *substantia nigra *(the most vulnerable area of midbrain in PD pathology) samples of three wild type mice were used for the electron microscopy investigation. Thirty micrographs were inspected for each tissue sample.

In order to in-depth scrutinize the notion of fundamental differential mitophagy performance in different mouse brain regions, we carried out a cellular ultra-structural examination to quantify the number of mitochondria undergoing mitophagy. There is no difference in either the total number of mitochondria (Figure 4A), or the number of mitochondria under fission or fusion among three brain regions investigated (data not shown). However, our histological examination using electron microscopy showed that a considerably higher amount of mitochondria were undergoing the process of mitophagy in normal mouse cortex tissue (2.28 ± 0.82 per μm^2) compared to normal striatum (0.85 ± 0.33 per μm^2, p = 0.009). The level of mitochondria undergoing mitophagy in cortex was also significantly higher than in substantia nigra (0.98 ± 0.39 per μm^2, p = 0.029). Moreover, compared to striatum, we observed significantly higher number of defective mitochondria in substantia nigra (2.05 ± 0.86 in SN vs. 0.98 ± 0.4 per μm^2 in striatum, p = 0.008). Figure 4 demonstrate the representative micrograph of cortex (7750×) showing recurrent incidences of mitophagic issue in all three wild type mouse brain regions.

## 4 Discussion

The manifestation of Parkinson's disease (PD) is initiated by a selective loss of dopaminergic neurons in the *substantia nigra pars compacta *(SN), a core complex in the midbrain. As dopamine is physiologically transported from the *substantia nigra *to the striatum by dopaminergic projections, this leads to a lack of dopamine in the striatum, which in turn causes prominent motor function disturbances [1]. In a sense, understanding of the selective vulnerability of dopamine neuron in *substantia nigra *could shed light on potential clues for PD therapeutic concept.

**Figure 5:**
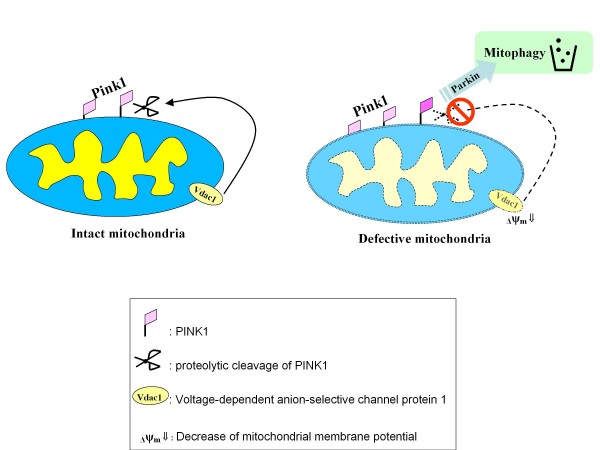
**Hypothetic mechanism of PINK1-Parkin mediated mitophagy according to the hypothesis raised by Narendra **[20,42]. The cellular mitochondria are governed by the quality control mechanism mitophagy, a form of autophagy in which defective mitochondria are selectively degraded by trafficking to lysosome. Left: PINK1 is a mitochondria located protein kinase with its enzymatic domain facing the cytosol. Expression of PINK1 on individual mitochondria is negatively regulated by Vdac1-dependent proteolysis to maintain low levels of PINK1 protein on intact mitochondria. Right: Loss of mitochondrial membrane potential (ΔΨ_m _⇓) leads to the accumulation of PINK1 on mitochondria. This gives Parkin the signal to recruit the PINK1-flagged mitochondria for mitophagy. This recruitment leads to the clearance of the defective organelle through the autophagosome formation.

Emerging evidences have been strengthening the link between the integrity of the neuronal mitochondria to PD. Here, mitophagy, a form of autophagy for selective degradation of defective mitochondria represent one of the key aspects [33,34]. As a major player in mitophagy mechanism, PINK1 is a mitochondrial protein kinase with its enzymatic domain facing the cytosol [14]. According to the hypotheses of Narendra and others, expression of PINK1 on individual mitochondria is negatively regulated by Vdac1-dependent proteolysis to maintain low levels of PINK1 protein on intact mitochondria (Figure 5, left). Loss of mitochondrial membrane potential, characterized by the Vdac1-downregulation, leads to the accumulation of PINK1 on mitochondria. This gives Parkin the signal to recruit the PINK1-flagged mitochondria to autophagosome for degradation (Figure 5) [20,35].

As typical post-mitotic cells, the neurons cannot distribute their damaged components to daughter cells, whereas cell death is unfavorable regarding tissue function [36]. This unique aspect of the neuron makes the finding of mitophagy-based cellular maintenance especially revealing concerning brain function perseverance [33]. Nevertheless, question remains regarding the formation of brain region specific neuronal loss in PD. With our current experimental setup, we aimed to interrogate the possible link between mitophagy functionality and the selective neuronal vulnerability in PD.

Among the available Parkinson's disease animal models, the mouse model with a loss-of-function mutation in PINK1 offers the most obvious link between mitophagy dysfunction and PD [21]. Unlike some other PD mouse models, the PINK1-KO mouse does not show dopaminergic neuron death. This was mirrored in our study by a missing observation of difference of oxidized protein levels in PINK1-KO in comparison to control. However, previous works have shown that loss of PINK1 causes clear-cut mitochondrial dysfunction and increased sensitivity of the neurons to oxidative stress, presumably through the blockage of mitophagy [21]. In the current study, different extents of proteomic shift was observed in different brain regions of our PD model, the PINK1-KO mice, with very little overlaps of protein expression alterations among brain regions. This reiterates the issue of brain region specificity in PD progression. Specifically, midbrain of PINK1-KO mice showed the highest number of non-redundant protein alterations. This correlates well with the higher vulnerability of this brain region that is specific for PD pathology.

Furthermore, our protein functional categorizations showed that there were significant down regulations of diverse mitochondrial energy metabolism pathways in the midbrain upon PINK1-KO (glycolysis, gluconeogenesis, pentose pathway, Krebs cycle, respiratory chain function). Lowered energy level can be considered as a general correlate to the accumulation of depolarized mitochondria, which is again associated with heightened oxidative stress [31,37].

We next investigated the oxidative protein damage patterns in three distinct brain regions with and without PINK1-KO. Our redox proteomic analysis showed that the cortex tissue was subjected to less extent of overall protein oxidative damage at both basal (wild type) and dynamic (under PINK1-KO) level: On the one hand, the Oxyblot staining experiments demonstrated minor extent of oxidative damage in the cortex tissue irrespective to the transgene. On the other hand, higher oxidative defense in cerebral cortex region was mirrored by the up-regulation of antioxidant-related proteins in our PD mouse model (glutathione synthetase and peroxiredoxin 1). Gluthatione is considered as the most important intracellular antioxidant. Upon the lack of glutathion, highly toxic hydroxyl radicals (OH·) will be generated though Fenton reaction. In sequence with this, the feedback up-regulation of glutathione synthetase has been considered as one of the earliest detectable changes in presymptomatic PD development [38]. Under the insult of PINK1-KO, higher expression of the oxidative protection protein glutathione synthetase was observed in cortex and striatum tissues of the PINK1-KO mice. Importantly, such phenomenon was not observed in midbrain tissues. Together, our results suggest that cortex, and to a lesser extent striatum, could be better protected against oxidative damage.

Most intrinsically, the observed drastically reduced Vdac1 protein level in midbrain tissue of the PINK1-KO mice offer us signs of the loss of mitochondrial membrane potential in the midbrain of PINK1-KO mice. Remarkably, this phenomenon was absent in striatum, whereas an up-regulation of Vdac1 proteins was observed in cortex tissue under PINK1-KO, which could indicate certain compensation mechanism in cortex tissues upon PINK1-KO. Drastically reduced Vdac1 protein level in midbrain tissue of the PD mouse model could reflect the brain region specific depolarization of mitochondria in PINK1-KO mice. Although such speculation would need direct investigations on mitochondrial membrane potential, this could suggest that mitophagy dysfunction induced by PINK1-KO led to a severe accumulation of non-potentiated mitochondria predominantly in the midbrain tissue [39].

To test this hypothesis of "built-in" brain-region specific mitophagy capacity in mouse, we conducted an electron microscopy study on wild type mice. Here, the normal mouse cortex tissue showed a 60% higher occurrence of mitophagic vacuoles compared to *substantia nigra*, whereas the amount of mitophagic vacuoles in striatum was comparable to that of *substantia nigra*. Even more notably, SN indeed contained intrinsically higher number of defective mitochondria in comparison to striatum. These observations ultimately validate the intrinsic differential mitophagic capacity in different brain regions.

The fact that PINK1 knockout induced mitophagy disturbance did not adversely influence the cortex and striatum tissues in terms of mitochondrial energy metabolism suggests some compensatory mechanism regarding cellular maintenance. In this respect, recent reports showed that autophagy and ubiquitination/proteasome pathways are two closely interwired cellular catabolic machineries [40,41]. We observed that two ubiqitination-related proteins were up-regulated in cortex and striatum tissues after PINK1-KO-induced mitophagy dysfunction. This implies that cortex and striatum tissues were able to respond to the mitophagy failure by stimulating the ubiquitin pathway as alternative catabolic mechanism, whereas midbrain did not. In the same scenario, the heat shock protein 70 (Hspa8), an important mediator of chaperone-mediated autophagy, was significantly up-regulated in cortex tissue, but not in striatum or midbrain under PINK1-KO. Together, this prompted us to suspect that the cerebral cortex tissue can compensate for mitophagic failure by stimulation of alternative catabolic process to rid the cell from oxidized proteins. Such capability could have increased the robustness of cortex tissue against oxidative damage.

In summary, using the PINK-KO mouse as a model, our hypothesis advanced in this work is that selective neuron vulnerability in Parkinson's disease could be co-determined by differential mitophagy capacity, differential oxidative protection, as well as differential feedback stimulation of alternative catabolic mechanisms in distinct brain regions. Our 2D-DIGE based study provides a starting point towards such advanced hypothesis, which will need to be scrutinized by subsequent investigations on mitochondrial membrane potential, metabolism functions and most importantly, direct studies on comprehensive mitotophagy pathway.

## Abbreviations

2DE: two-dimensional protein electrophoresis; 2D-DIGE: two dimensional difference gel electrophoresis; KO: knock out mouse model; PD: Parkinson's disease; SEM: standard error of mean; SN: substantia nigra.

## Competing interests

The authors declare that they have no competing interests.

## Authors' contributions

MD performed the proteomic experiments and wrote the manuscript. TK generated the transgenic mice and prepared the tissue samples. GN and AK performed the mass spectrometric analysis. JS co-designed the experiments. CZ performed the statistical analysis. JK co-designed the experiments. LM co-designed the experiments, coordinated the project and wrote the manuscript. All authors read and approved the final manuscript.
